# FIVE REALITIES: A TYPOLOGY OF CAREGIVERS OF PERSONS WITH DEMENTIA AND THEIR INTERVENTION OUTCOMES

**DOI:** 10.1093/geroni/igad104.1228

**Published:** 2023-12-21

**Authors:** Sato Ashida, Freda Lynn, Lena Thompson, Maria Donohoe, Haley Schneider, Kristine Williams

**Affiliations:** University of Iowa College of Public Health, Iowa City, Iowa, United States; University of Iowa, Iowa City, Iowa, United States; University of Minnesota, Duluth, Minnesota, United States; University of Iowa College of Public Health, Iowa City, Iowa, United States; University of Iowa, Iowa City, Iowa, United States; University of Kansas Medical Center, Kansas City, Kansas, United States

## Abstract

Caregivers of persons living with dementia report wide-ranging lived experiences, including feelings of burden and frustration but also positive outcomes. This study uses novel survey data to describe distinct types of caregiving experiences from a holistic perspective. We then explore the extent to which responses to a caregiver intervention are contingent on experience type. Using baseline data from a randomized trial, we use the k-means clustering algorithm on eight caregiver perceptions (adversity: burden, anxiety, network unmet-expectations, and network stress; positivity: positive aspects of caregiving, self-efficacy accessing services, caregiving preparedness, and network support) to identify experience types. Differential responses to intervention at 3-months by typology were assessed using OLS regression methods. Eighty-one caregivers were partitioned into five latent classes: Thriving (low adversity, high positivity); Deeply Struggling (high adversity, low positivity); Struggling but feeling prepared; Struggling and unprepared; No Plans (unprepared but not anxious). Attrition was highest among struggling groups and lowest among thrivers. Caregivers who are “Struggling but feeling prepared” (p=0.01) or “Struggling and unprepared” (p=0.03) showed significant increase in self-efficacy to use community services at 3-months. The latter group also showed a significant increase in knowledge about community services (p=0.03). Network stress at 3-months increased significantly more for the control than intervention group (p=0.03); by cluster, group differences were most pronounced among “Deeply struggling” (p=0.04) and “No Plans” (p=0.05). Types of caregiving experiences may determine caregiver participation in programs and their response to programs intended to support caregivers. Research and practice should focus on identifying strategies that fit caregiver experiences.

